# Biochemical and Structural Analysis of a Glucose-Tolerant β-Glucosidase from the Hemicellulose-Degrading *Thermoanaerobacterium saccharolyticum*

**DOI:** 10.3390/molecules27010290

**Published:** 2022-01-04

**Authors:** In Jung Kim, Uwe T. Bornscheuer, Ki Hyun Nam

**Affiliations:** 1 Department of Biotechnology and Enzyme Catalysis, Institute of Biochemistry, University of Greifswald, 17489 Greifswald, Germany; ij0308@korea.ac.kr (I.J.K.); uwe.bornscheuer@uni-greifswald.de (U.T.B.); 2 Department of Life Science, Pohang University of Science and Technology, Pohang 37673, Korea; 3 POSTECH Biotech Center, Pohang University of Science and Technology, Pohang 37673, Korea

**Keywords:** β-glucosidase, Bgl, glycoside hydrolase, cellulose degradation, glucose tolerance, Tris inhibition, *Thermoanaerobacterium saccharolyticum*

## Abstract

β-Glucosidases (Bgls) convert cellobiose and other soluble cello-oligomers into glucose and play important roles in fundamental biological processes, providing energy sources in living organisms. Bgls are essential terminal enzymes of cellulose degradation systems and attractive targets for lignocellulose-based biotechnological applications. Characterization of novel Bgls is important for broadening our knowledge of this enzyme class and can provide insights into its further applications. In this study, we report the biochemical and structural analysis of a Bgl from the hemicellulose-degrading thermophilic anaerobe *Thermoanaerobacterium saccharolyticum* (TsaBgl). TsaBgl exhibited its maximum hydrolase activity on *p*-nitrophenyl-β-d-glucopyranoside at pH 6.0 and 55 °C. The crystal structure of TsaBgl showed a single (β/α)_8_ TIM-barrel fold, and a β8-α14 loop, which is located around the substrate-binding pocket entrance, showing a unique conformation compared with other structurally known Bgls. A Tris molecule inhibited enzyme activity and was bound to the active site of TsaBgl coordinated by the catalytic residues Glu163 (proton donor) and Glu351 (nucleophile). Titration experiments showed that TsaBgl belongs to the glucose-tolerant Bgl family. The gatekeeper site of TsaBgl is similar to those of other glucose-tolerant Bgls, whereas Trp323 and Leu170, which are involved in glucose tolerance, show a unique configuration. Our results therefore improve our knowledge about the Tris-mediated inhibition and glucose tolerance of Bgl family members, which is essential for their industrial application.

## 1. Introduction

Biomass-derived fuels are believed to play major roles in the global energy future, and technological development driving down costs are important for competing with petroleum and other alternative fuels [1]. Lignocellulose is a promising biomass feedstock that can be utilized for the sustainable production of biofuels and biochemicals [2]. In lignocellulose-derived biorefineries, enzymatic hydrolysis is required to obtain fermentable sugars, which microorganisms can convert into fuels and value-added chemicals [3]. However, cellulose, a polymer consisting of glucose that is contained in lignocellulose, is inherently recalcitrant for enzymatic degradation and densely packed and arranged, forming a highly crystalline and insoluble structure [4]. Such a microfibril structure prevents cellulolytic enzymes from accessing the β-1,4-glycosidic bonds in cellulose [4]. Consequently, natural microbial systems require the cooperative or synergistic action of different hydrolytic cellulases, such as endoglucanase (EG), cellobiohydrolase (CBH), and β-glucosidase (Bgl), for the saccharification of cellulose. EGs randomly attack the internal bonds of cellulose polymer chains at amorphous regions, producing oligosaccharides with varying degrees of polymerization. CBHs, also referred to as exocellulases, cleave cellulose chains either from their reducing (CBH 1) or nonreducing ends (CBH 2) in a progressive manner to yield cellobiose as the major product. This cellobiose is then converted into glucose by Bgl, making Bgl an essential enzyme in the final step of cellulose degradation [5,6,7,8,9].

Retaining Bgls have been found in the glycoside hydrolase (GH) families GH1, GH3, GH5, GH9, GH30, and GH116 of the CAZy database [10]. The GH1 family contains two Bgl homologs, BglA and BglB; both show hydrolase activity on cellodextrin, but differ in their quaternary structure and substrate specificity [11]. BglA is an unusual octameric cellobiase, whereas BglB is a monomeric enzyme that hydrolyzes cellobiose and cellodextrins with a high degree of polymerization [11,12,13,14,15]. Bgl cleaves β-1,4-glycosidic linkages in disaccharides and glucose-substituted polysaccharides and is involved in the utilization of polysaccharides as an energy source in cells [16,17]. Moreover, Bgl is widely applied in lignocellulosic biomass degradation [18]. Although various Bgls have been functionally and structurally analyzed, numerous Bgls, which are highly attractive for industrial applications, remain uncharacterized.

Thermophilic anaerobic bacteria include species with the natural ability to digest and ferment polysaccharides that constitute lignocellulosic biomass [19,20]. *Thermoanaerobacterium saccharolyticum* is a hemicellulose-degrading thermophilic anaerobe and a biological catalyst for the conversion of cellulosic biomass to ethanol [21]. This organism grows at temperatures ranging from 30 to 66 °C and at pH values ranging from 3.85 to 6.35 [21]. This species ferments various carbohydrates, such as glucose, cellobiose, xylan, xylose, starch, arabinose, mannose, and galactose, but cannot degrade crystalline cellulose [22]. Various thermophilic enzymes, which can be applied in industry, have been isolated and characterized from *T. saccharolyticum*, including endoxylanase [23], β-xylosidase [24], glucuronidase [25], and amylopullanase [26]; however, Bgl, the terminal enzyme of the cellulose degradation process, is still unknown.

In this study, we report the biochemical and structural analysis of Bgl from *T. saccharolyticum* (TsaBgl). We investigated the substrate specificity, optimum temperature and pH, and thermal stability of TsaBGl and the effects of metals on this enzyme. A high-resolution crystal structure of TsaBGl was determined at 1.7 Å, showing the binding of the inhibitor Tris to the active site. Moreover, a titration experiment of TsaBgl using Tris and glucose molecules was performed. Our results showed that the favorable properties of TsaBgl could be used in the field of biotechnology and provide useful information to broaden our structural knowledge of the Bgl family.

## 2. Results

### 2.1. β-Glucosidase Activity of TsaBgl

**Figure 1:**
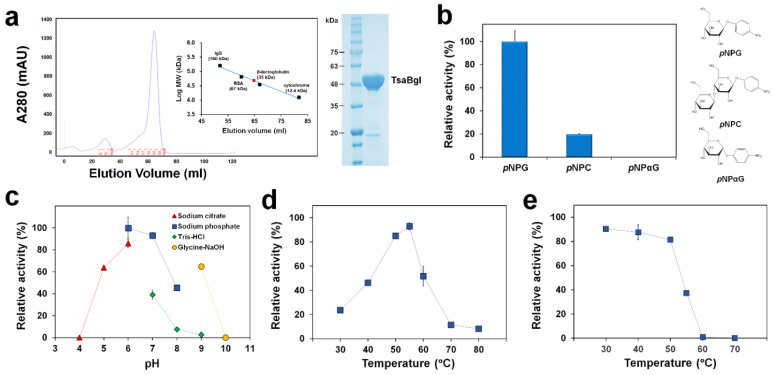
Characterization of the β-glucosidase activity of TsaBgl. (**a**) Elution profile of TsaBgl on gel filtration chromato-graphy and purified protein. (**b**) Enzyme activity of TsaBgl towards various *p*-nitrophenyl substrates at 55 °C in 100 mM sodium phosphate (pH 6.0). (**c**) Enzyme activity of TsaBgl at various pH, ranging from pH 4 to 10, at 55 °C. (**d**) Enzyme activity of TsaBgl at various temperatures ranging from 30 to 80 °C in 100 mM sodium phosphate buffer (pH 7.0). Relative activity in panels (**b**–**d**) is defined as the enzyme activity relative to that obtained at pH 6.0 (100 mM sodium phosphate) and 55 °C with *p*NPG, set as 100%, with a specific activity of 23.6 U/mg. (**e**) Thermal stability of TsaBgl. The enzyme was pre-incubated at 30, 40, 50, 60, and 70 °C for 10 min without substrate, after which residual activities were measured under the standard conditions as detailed in the Materials and Methods section. The activity without pre-incubation was set as 100%, where the specific activity was 23.6 U/mg, and residual activities were calculated as its percentage. Error bars represent the standard deviations of three independent experiments.

TsaBgl, codon-optimized for *E. coli* host expression, was overexpressed in *E. coli* BL21 (DE3). The protein was eluted at a molecular mass position of approximately 50 kDa during size exclusion chromatography, which was almost identical to the theoretical molecular weight of TsaBgl (51.728 kDa), indicating that TsaBgl was a monomer in solution (Figure 1a). The enzyme activity and substrate specificity of purified TsaBgl were determined using various *p*-nitrophenyl glycosides (*p*NPG, *p*NPC, and *p*NPαG) as substrates. *p*NPG and *p*NPC were selected based on the fact that Bgls often possess hydrolyzing activity towards soluble cellodextrins, including cellobiose and cellotriose. In addition, α-bonded- *p*NPαG was chosen to examine the preferred linkage type in TsaBgl. Our results show that TsaBgl exhibited the highest hydrolase activity on the artificial substrate *p*NPG with a specific activity of 23.6 U/mg. Moreover, the relative hydrolase activity of TsaBgl toward *p*NPC was 19.71% of that toward *p*NPG; however, no enzyme activity was observed on *p*NPαG (Figure 1b).

Next, the optimal temperature and pH for TsaBgl enzyme activity were investigated using *p*NPG as a substrate. For both profiles, the activity at pH 6.0 (100 mM sodium phosphate) and 55 °C was defined as 100%, where the specific activity was 23.6 U/mg. The hydrolase activity of TsaBgl was investigated in the pH range of 4.0–10.0 using a four buffer system (Figure 1c). The maximum enzyme activity of TsaBgl was obtained at pH 6.0, and at pH 5.0, 7.0, and 9.0, the relative activity was still higher than 60%. Additionally, the relative activity was nearly zero at pH 4.0 and 10.0. Although generally approved, such a system using different buffers can sometimes cause a significant deviation in activity values at the same pH (but in different buffers), especially when the target enzyme is sensitive to a certain buffer component. Notably, the enzyme activities determined from pH 7.0 to 9.0 using Tris-HCl buffer were much lower than those obtained in sodium phosphate and glycine-NaOH buffers (Figure 1c). This implied that the Tris molecule could act as a competitive inhibitor of TsaBgl (see below). Therefore, Tris would not be a good buffer for application of TsaBgl; other buffers, such as phosphate-based buffers, should be used instead, in the same pH range. However, the final concentration of Tris at 1 mM, derived from the storage buffer (10 mM Tris-HCl with 200 mM NaCl; pH 8.0), in our enzyme assays did not significantly affected the activity of TsaBgl compared to that in a reaction without 1 mM Tris (*p* < 0.05) (Figure S1).

The enzyme activity of TsaBgl was then determined over a temperature range of 30–70 °C (Figure 1d). Maximum enzyme activity was obtained at 55 °C, and the relative activity was still 91% at 50 °C. However, the activity dropped sharply at 60 °C (relative activity: ~55%), and at 70 °C, only 12% of the relative activity was left. In addition to the reaction rate, it is important to examine the enzyme stability in response to heat when considering its industrial applications. We found that after 10 min of incubation at 30, 40, and 50 °C, more than 80% of the activity of TsaBgl was retained (Figure 1e). However, the residual activity dramatically decreased to 37% at 55 °C, and only a little activity remained at 60 °C. These results indicated that, although the activity of TsaBgl was highest at 55 °C, the structure was not sufficiently stable to maintain its conformation and activity at this temperature.

The effects of metal ions on TsaBgl activity were also assessed (Table 1). The specific activity without any metal ion was 25.9 U/mg. As a result, no metal ion distinctively stimulated enzyme activity. In contrast, activity was markedly reduced in the presence of 1 mM Cd^2+^, Zn^2+^, Cu^2+^, and Fe^2+^ compared with that in the absence of any metal. The inhibition trend was as follows: Cu^2+^ > Zn^2+^ > Cd^2+^ and Fe^2+^, with relative activities of 8.7%, 25.2%, 68.2%, and 71.2%, respectively (Table 1).

Kinetic parameters for TsaBgl were obtained with respect to 0 to 10 mM *p*NPG under the standard conditions, wherein *K_m_*, *k_cat_*, and *k_cat_*/*K_m_* were 0.36 mM, 18.62 s^−1^, and 50.99 mM^−1^ s^−1^, respectively.

### 2.2. Overall Structure of TsaBgl

To better elucidate the molecular function of TsaBgl, we determined the crystal structure of TsaBgl at 1.70 Å resolution (Table 2). Crystals of TsaBgl belonged to the orthorhombic space group P2_1_2_1_2_1_ with one molecule per asymmetric unit. The final R_work_ and R_free_ of the TsaBgl model were 14.10% and 18.23%, respectively.

**Figure 2:**
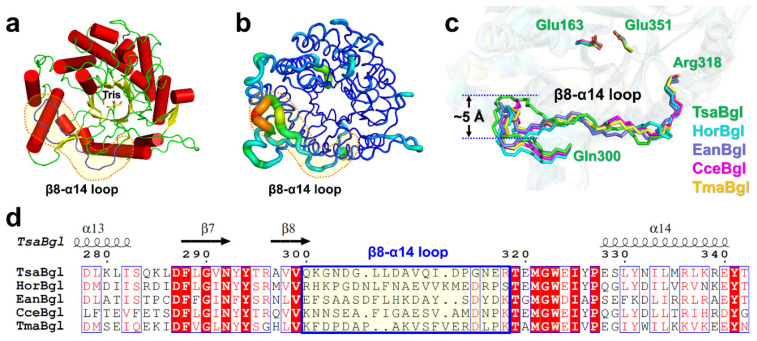
Crystal structure of TsaBgl and comparison with other Bgls. (**a**) Cartoon representation of the TsaBgl structure with a Tris molecule bound to the active site in the center of the TIM-barrel fold. (**b**) B-Factor putty representation of TsaBgl. The β8-α14 loop is more flexible (green to red) than other regions (blue). (**c**) Close-up view of the superimposition of β8-α14 loop region of TsaBgl with that of HorBglB (PDB code: 4PTX), EanBglB (5DT7), CceBglB (3AHX), and TmaBgl (2J79). (**d**) Partial sequence alignments of TsaBgl (UniProt code: I3VXG7) with HorBglB (B8CYA8), EanBglB (K0A8J9), CceBglB (Q53EH2), and TmaBgl (Q08638).

TsaBgl contained 16 α-helices and 13 β-strands, forming a single (β/α)_8_ TIM-barrel fold with dimensions of 55 × 55 × 50 Å (Figure 2a). During the structure refinement, strong Fo-Fc electron density was observed in the active site pocket corresponding to the Tris molecule of the buffer solution (Figure S2), which was characterized as an inhibitor in our biochemical study (Figure 1c). Interactions between the N-terminus of TsaBgl (Asp3, Phe4, Ser5, Lys6, and Phe8) and its C-terminus (Arg442, Thr443, and Ieu444) were observed (Figure S3), and these interactions were involved in protein stability [27]. The conserved active site containing the crucial residues Glu163 (proton donor) and Glu351 (nucleophile), which are involved in the acid-base mechanism, is located in the center of the TIM-barrel (Figure 2a). Temperature factor analysis showed that the core of the TIM-barrel of TsaBgl was highly rigid, whereas the loop between the β8-strand and α14-helix (named the β8-α14 loop) showed structural flexibility and was ~20 Å away from the substrate-binding entrance (Figure 2b). In particular, residues between Gln300 and Arg318 in the β8-α14 loop (B-factor value: 33.92 Å^2^) exhibited a considerably higher B-factor than the whole protein (17.37 Å^2^).

The results of the structural homology search showed that TsaBgl shared high structural similarities with Bgls from *Halothermothrix orenii* (HorBglB, PDB code: 4PTX, Z-score: 63.3, sequence identity: 52.32%), *Exiguobacterium antarcticum* (EanBglB, 5DT7, 63.1, 50.78%), *Clostridium cellulovorans* (CceBglB, 3AHX, 61.9, 53.23%), and *Thermotoga maritima* (TmaBglB, 2J79, 61.7, 47.78%). Superimposition of the TsaBgl structure with those of the homologous Bgls showed high similarity, with r.m.s. deviations of 0.49–0.61 Å (Figure S4). Additionally, pronounced differences in the conformations of their β8-α14 loops were also observed (Figure 2c). These loop regions did not have sequence similarity with each other (Figure 2d).

### 2.3. Tris-Binding at the Active Site of TsBgl

**Figure 3:**
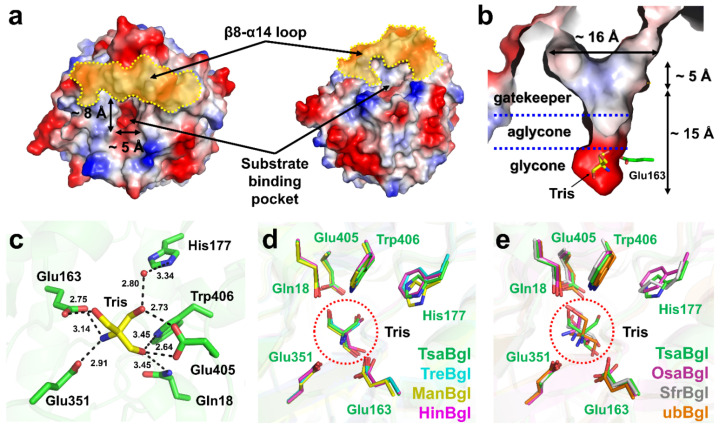
Substrate-binding pocket and active site of TsaBgl. (**a**) Electrostatic potential surface of the substrate-binding site of TsaBgl. The β8-α14 loop is indicated by a yellow dotted line. (**b**) Substrate binding channel of TsaBgl. Tris is bound to the glycone region of TsaBgl. (**c**) Interactions between the Tris molecule and the residues of the substrate-binding site in TsaBgl. The interacting residues Glu163 and Glu351 are catalytic residues for Bgl activity. (**d**) Similar Tris-binding configurations of TsaBgl with TreBgl (PDB code: 3AHY), ManBgl (3W53), and HinBgl (4MDO). (**e**) Different Tris-binding configuration of TsaBgl and OsaBgl (4RE2), SfrBgl (5CG0) and ubBgl (6IER).

The substrate-binding pocket of TsaBgl, including the active site, is located in the center of the TIM-barrel fold (Figure 3a). The part of the β8-α14 loop pointing toward the substrate-binding pocket and the part of the entrance of the substrate-biding pocket in the vicinity of the β8-α14 loop forms a hydrophobic surface with an area of approximately 5 × 16 Å (Figure 3b). In particular, the area and shape of this hydrophobic surface depended on the conformation of the β8-α14 loop. The size of the entrance of the substrate-binding pocket was approximately 5 × 8 Å (Figure 3a), and the distance from the entrance surface to the active site was approximately 15 Å (Figure 3b). Thus, linear sugar substrates, such as cellobiose and *p*NPG, could access and intrude into the substrate-binding site, whereas bulky sugar substrates, such as branched sugars, could not pass through the gatekeeper region in the substrate binding site.

The active site residues Glu163 and Glu351 in TsaBgl are absolutely conserved among the members of the Bgl family (Figure S5). Glu163 and Glu351 act as an acid/base catalyst and nucleophile, respectively, during the catalytic mechanism [28]. The glycone region has a negatively charged surface, and one Tris molecule was bound to the active site (Figure 3b). The O1 atom of Tris interacts with the OE1 atoms of Glu163 (2.75 Å). The O2 atom of Tris interacts with the OE2 atom of Glu405 (2.73 Å) and the NE2 atom of His177 via a water bridge. The O3 atom of Tris interacts with the OE1 atom of Glu405 (2.64 Å), the NE2 atom of Gln18 (3.45 Å), and the NE1 atom of Trp398 (3.45 Å) (Figure 3c). The N atom of Tris interacts with the OE2 atom of Glu163 (3.14 Å) and the OE1 atom of Glu351 (2.91 Å). All amino acids interacting with Tris are conserved in the Bgl family (Figure S5).

In the Protein Data Bank, we found eight crystal structures of Tris-bound Bgls: Bgl from *Trichoderma reesei* (TreBgl, PDB code: 3AHY) [29], *Oryza sativa* (OsaBgl, 3PTK) [29], *Micrococcus antarcticus* (ManBgl, 3W53) [30], *Humicola insolens* (HinBgl, 4MDO) [31], *Oryza sativa* subsp. indica (Rice) (OsaBgl2, 4RE2 and 4RE3) [32], *Spodoptera frugiperda* (SfrBgl, 5CG0) [33], and from an uncultured bacterium (ubBgl, 6IER) [34,35]. The binding configuration of the Tris molecule in the active site of TsaBgl was similar to those in the structures TreBgl, ManBgl, and HthBgl (Figure 3d), whereas the structures OsaBgl, SfrBgl, and ubBgl showed distinct binding configurations of Tris (Figure 3e). 

On the other hand, in the structures OsaBgl2, the Tris molecule was located in the binding channel or at the surface of the TIM-barrel.

### 2.4. Titration of Tris and Glucose on TsaBgl

**Figure 4:**
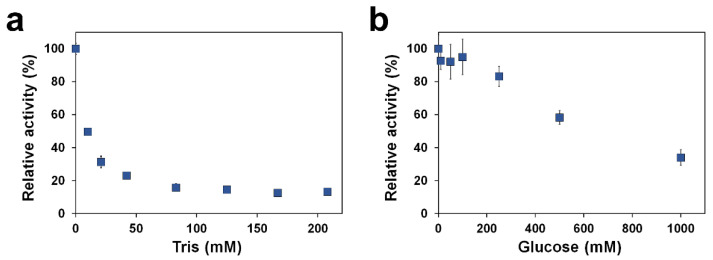
Enzyme activity of TsaBgl under various concentrations of (**a**) Tris (pH 7.0 with 100 mM sodium phosphate) and (**b**) glucose (pH 6.0 in 100 mM sodium phosphate) was performed at 55 °C using *p*NPG as substrate. Relative activity (%) in each panel represents the enzyme activity relative to that obtained under conditions lacking any inhibiting molecule (i.e., Tris or glucose), defined as 100%, where the specific activities were 22.7 and 19.1 U/mg, respectively. Error bars represent the standard deviations of three independent experiments.

Glucose produced by the Bgl-catalyzed decomposition of cellobiose is known to act as a product inhibitor [35]. This is a potential problem preventing the production efficiency in industrial applications. To investigate the binding mode of glucose, we soaked TsaBgl crystals with various concentrations of glucose for more than 1 h before collection of diffraction data. The results show that no electron density corresponding to glucose was observed in the active site of TsaBgl, but a Tris molecule bound to the active site in the same way as in the native crystal structure of TsaBgl. Therefore, we assumed that glucose might have a lower binding affinity for the active site of TsaBgl than Tris. To provide experimental evidence for this, we performed an analysis of hydrolysis inhibition of TsaBgl through titration experiments on Tris and glucose molecules. Relative activity (%) in each panel represented the enzyme activity relative to that obtained under the condition lacking any inhibitory molecule (i.e., Tris or glucose), the latter being defined as 100%. Specific activity in the absence of Tris or glucose was 19.1 or 22.7 U/mg, respectively. The enzyme activity of TsaBgl was significantly inhibited by the Tris molecule (Figure 4a). A Tris concentration of 10 mM reduced the enzyme activity by half. In the presence of 80 mM Tris, only 15% of the activity remained. In contrast, TsaBgl maintained its full activity, even at glucose concentrations of up to 100 mM, and in the presence of 200 mM glucose, 80% of the maximum activity (without glucose) still remained (Figure 4b). The inhibition concentration of glucose that reduced the activity of TsaBgl by half was approximately 650 mM; however, even at higher glucose concentrations of up to 830 mM, the enzyme still showed a relative activity of 34%. Therefore, the enzyme was more sensitive to Tris than to glucose, and its activity sharply declined, even at low concentrations of Tris. The observation that TsaBgl maintained its activity, even in the presence of such high glucose concentrations, indicates that TsaBgl is a glucose-tolerant Bgl. Taken together, these findings demonstrate that the Tris molecule acts as inhibitor of TsaBgl and that TsaBgl belongs to the glucose-tolerant Bgl family.

## 3. Discussion

Bgls are essential terminal enzymes of the cellulase system and attractive targets for lignocellulose-based biotechnological applications. To discover a novel Bgl and broaden the knowledge of this enzyme class at the molecular level, we performed biochemical and structural analyses of Bgl from the hemicellulose-degrading thermophilic anaerobe *T. saccharolyticum*. The growth pH and temperature of the *T. saccharolyticum* strain were 3.85–6.35 and 30–66 °C, respectively. Purified recombinant TsaBgl showed high activity at pH 6.0 and 50–55 °C, but the activity decreased to less than 60% at other pH and temperature values. In addition, TsaBgl showed the highest enzyme activity at 55 °C, although thermostability experiments revealed that the residual activity was reduced by 37% after 10 min. As a result, the in vitro enzymatic activity of purified TsaBgl was not as consistent over a broad pH and temperature range as the growth of the strain.

A previous study had classified the Bgl family into five clades: Clade I (mainly mesophilic bacteria), Clade II (GH1 Bgl from fungi), Clade III (GH3 Bgl from bacteria), Clade IV (GH3 Bgl from fungi), and Clade V (thermophile or Bacillus GH1 Bgl) [36]. Taxonomic analysis showed that TsaBgl belongs to Clade V and has close evolutionary relationships with *Thermoanaerobacterium* *thermosaccharolyticum* (Figure S6). In addition, TsaBgl exhibits high amino acid sequence identity of 87.2% (similarity of 94%) to Bgl from *Thermoanaerobacterium* *thermosaccharolyticum* (named TthBgl). Despite the close relationship between TsaBgl and TthBgl, there are several notables differences in their enzymatic activity. Specifically, the optimum temperature of TsaBgl and TthBgl for enzyme activity are 55 and 70 °C, respectively. Moreover, the activity of TsaBgl was not affected by Mn^2+^ and inhibited by Fe^2+^ while the activity of TthBgl was activated by both Mn^2+^ and Fe^2+^. Such a discrepancy may arise from a difference in the reaction buffer between the two studies (sodium phosphate vs. imidole-potassium). Since phosphate often forms an insoluble complex with metal ions, in our study, effects of Fe^2+^ and Mn^2+^ might have been either insignificant or inhibitory in phosphate buffer. Furthermore, in the current study, TsaBgl was incubated with metal ions in the presence of the substrate while TthBgl was pre-incubated with metal ions prior to substrate addition. Since the latter had a longer time for direct interaction with metal ions that might explain the discrepancy between the two enzymes regarding activities in the presence of Mn^2+^.

Kinetic parameters of TsaBgl were compared to those of other Bgls belonging to GH family 1. The parameters of different Bgls showed distinct values (Table 2). The *K_m_* value of TsaBgl, which represents binding affinity, was slightly lower or comparable to that of other Bgls, indicating for TsaBgl, in general, to have a higher or similar binding affinity relative to GH1 Bgls. While the *k_cat_* values, the turnover number of substrate, were similar across TsaBgl, EanBgl, and CceBgl, TsaBgl showed higher values than TrBgl2 and NkoBgl by approximately 3 and 13 fold, respectively, whereas this value was 24-fold lower than that of TmaBglA. The overall catalytic efficiency of TsaBgl for *p*NPG (*k_cat_/K_m_*) compared to that of EanBglA was higher than those reported for TreBgl2 and NkoBgl, but 7- and 24-fold lower than those reported for CceBglA and TmaBglA, respectively.

The catalytic efficiency (*k_cat_/K_m_*) of TsaBgl was similar to that of EanBglA, another glucose-tolerant GH1, but much higher than those of TrsBgl2 and NkoBgl (Table 3), whose active sites are known to be occupied and actions are inhibited by Tris, like in TsaBgl reported in our study. Conversely, the catalytic efficiency of CceBgl and TmaBglA were much higher than those of TsaBgl. In particular, TmaBglA was exceptionally superior in terms of catalytic efficiency, which might be owing to its hyperthermostability. However, to the best of our knowledge, none of the two enzymes had been reported to be glucose-tolerant and thus, TsaBgl with glucose-tolerance could be a good candidate for industrial application.

The sequence similarity between TsaBgl and other structurally known Bgls was less than 53%. Although the overall TIM-barrel fold was similar among all Bgls, the β8-α14 loop of TsaBgl showed a unique conformation. As loops near the substrate-binding site of Bgl can be flexible during substrate binding [39], we assumed that the β8-α14 loop of TsaBgl may adopt another conformation during substrate recognition. Moreover, the unique conformation of the β8-α14 loop of TsaBgl structurally supported the suggestion that variations in the substrate recognition loop of the Bgl family could be varied.

Tris molecules often act as inhibitors to reduce the hydrolytic enzyme activity of Bgl [40,41,42], and this molecule is presumed to mimic the substrate because it is rich in hydroxyl groups [11,30]. Our biochemical results showed that the enzyme activity of TsaBgl was also reduced by Tris. In the current study, TsaBgl was purified and stored in Tris buffer. When the TsaBgl solution stored in 10 mM Tris buffer was diluted with other buffers, such as phosphate and glycine, the enzyme activity was increased, indicating that Tris could be released from TsaBgl in the presence of low concentrations of Tris. Analysis of the crystal structure of TsaBgl showed that one Tris molecule is bound to the glycone site and interacted with Glu163 and Glu351, which are involved in the hydrolytic activity of TsaBgl. The binding configuration of Tris bound to TsaBgl was almost identical to those of TreBgl, ManBgl, and HthBgl (Figure 3d), but different from those of OsaBgl, SfrBgl, and ubBgl (Figure 3e). Additionally, among the Bgl structures deposited in the PDB, some structures showed that Tris did not bind to the active site despite the use of Tris buffer during purification or crystallization; these Bgls are expected to have no high binding affinity with Tris. Collectively, our findings suggest that the inhibitory effects of Tris on enzyme activity varied for each Bgl. These results are meaningful because Tris may limit the potential industrial applications of some Bgls. On the other hand, if the active site is occupied by Tris, the actual substrate cannot directly access the substrate-binding site, thereby reducing enzyme activity because additional steps are required to remove Tris from the substrate-binding site. Therefore, when measuring the enzyme activities of Bgls, buffers other than Tris (e.g., phosphate-based buffers) should be tested in the same pH range; this could be important for optimizing enzyme activity for industrial applications.

During the production of biofuels or biochemicals derived from lignocellulosic biomass, a high titer of glucose is generated. In addition, glucose, the end-product of the catalytic reaction of Bgl, can inhibit enzyme activity and often poses a limitation on the use of Bgls in industrial applications [35]. To better understand glucose recognition of TsaBgl, we performed a soaking experiment to bind glucose to TsaBgl; however, in all crystal structures, Tris, not glucose, was bound to the active site, indicating that glucose had lower binding affinity than Tris. This result is consistent with a titration experiment for TsaBgl using Tris and glucose, which showed that TsaBgl belonged to the glucose-tolerant Bgl family.

Depending on the effects of glucose on enzyme activity, Bgls can be divided into three groups [43]: (1) those that are strongly inhibited by low concentrations of glucose (including most Bgls); (2) those that are resistant to low levels of glucose, but inhibited by high glucose; and (3) those that are stimulated by low glucose levels and inhibited by high glucose levels. TsaBgl belongs to the second group and showed activity that was resistant to glucose at concentrations of up to 0.3 M, but inhibited (50% of original activity) by 0.65 M glucose.

Previous studies have identified several key amino acids that influence glucose tolerance, most of them at the substrate entrance site [44,45,46]. In particular, hydrophobic interactions of tryptophan and leucine resides contribute to relieving enzyme inhibition by imposing constraints at the gatekeeper region that limit the access of glucose to the glycone site [31,44]. These two residues are conserved in TsaBgl (Figure S5).

**Figure 5:**
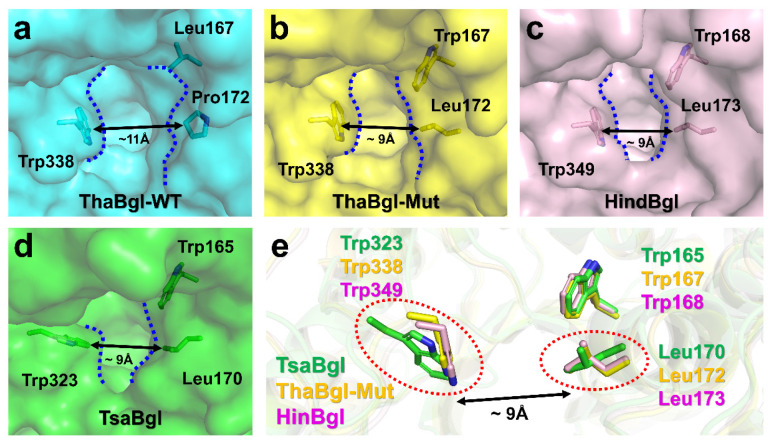
Comparison of the active site pocket of (**a**) wild-type ThaBgl (PDB code: 5BWF) and (**b**) engineered ThaBgl (6EFU), (**c**) HinBgl (4MDO), and (**d**) TsaBgl. (**e**) Superimposition of conserved Trp and Leu residues involved in the glucose tolerance of TsaBgl, ThaBgl-Mut, and HinBgl.

To understand the structural basis of glucose tolerance better, we compared the TsaBgl structure with those of the previously reported glucose-tolerant engineered *Trichoderma harzianum* Bgl (ThaBgl) and the high glucose-tolerant *Humicola insolens* Bgl (HinBgl). TsaBgl showed amino acid sequence identities of 38.0 and 36.0% with ThaBgl-Mut and HinBgl, respectively. Structural superposition of ThaBgl-Mut and HinBgl to TsaBgl showed r.m.s. deviation of 0.723 and 0.678 Å, respectively. In a previous study of ThaBgl, double amino acid replacements L167W/P172L in wild-type ThaBgl (ThaBgl-WT) dramatically increased the properties of glucose-tolerant ThaBgl (ThaBgl-Mut) [18]. The catalytic efficiency parameter (*k_cat_/K_m_*) of ThaBgl-Mut (7.85 ± 0.22 s^−1^/0.41 ± 0.30 mM) was approximately 5-fold higher than that of ThaBgl-WT (4.92 ± 0.54 s^−1^/1.17 ± 0.66 mM). In the crystal structure, ThaBgl-WT showed a wider aglycone channel measuring ~11 Å in width between Trp338 and Pro172 (Figure 5a), whereas ThaBgl-Mut showed a narrower aglycone channel measuring ~9 Å between Trp338 and Leu172 [18] (Figure 5b). This active-site topology is similar to high glucose-tolerant HinBgl [31], which exhibited an aglycone channel width of ~9 Å between Trp349 and Leu173 (Figure 5c). These results suggested the superb tolerance to high glucose concentrations owing to the reduced distance between the two sides of the active site entrance channel [18]. Additionally, TsaBgl also harbors a narrow aglycone channel of ~9 Å between Trp323 and Le170 (Figure 5d), similar to ThaBgl-Mut and HinBgl; however, the configuration of Trp and Leu residues are different (Figure 5e). To better understand the structural basis for glucose tolerance of TsaBgl, further studies are needed to obtain the Tris-free or other molecule binding state of TsaBgl.

In conclusion, our biochemical and structural analysis of TsaBgl is expected to improve our understanding of the Bgl family and provides new insights into the utilization of Bgl for industrial applications.

## 4. Materials and Methods

### 4.1. Protein Preparation

A construct containing codon-optimized full-length TsaBgl (accession number: MW655874) with an N-terminally fused hexahistidine-tag was synthesized and cloned into the pBT7 vector (Bioneer, Korea). Recombinant DNA was transformed into *Escherichia coli* BL21 (DE3). Cells were grown at 37 °C in LB medium with 50 mg/mL ampicillin until reaching an OD_600_ of 0.4. Protein expression was induced with 0.5 mM isopropyl-d-1-thiogalactopyranoside, and the cells were cultured at 18 °C for 18 h. After cell harvesting by centrifugation, the cells were resuspended in lysis buffer containing 50 mM Tris-HCl (pH 8.0), 200 mM NaCl, and 20 mM imidazole. The cells were lysed by sonication on ice, and cell debris was removed by centrifugation at 18,894× *g* for 30 min. The supernatant was loaded onto a Ni-NTA column (Qiagen, Valencia, CA, USA). The column was washed and equilibrated with lysis buffer, and the protein was subsequently eluted with a buffer containing 50 mM Tris-HCl (pH 8.0), 200 mM NaCl, and 300 mM imidazole. To remove the N-terminal hexahistidine-tag, 10 units of thrombin (Sigma Aldrich, St. Louis, MO, USA) were added per milligram of TsaBgl fraction and incubated overnight at 20 °C. The protein was concentrated using a Centricon filter (Merck Millipore, Burlington, MA, USA; cut-off: 30 kDa), applied onto a Sephacryl-100 column (GE Healthcare, Chicago, IL, USA), and eluted with a buffer containing 10 mM Tris-HCl (pH 8.0) and 200 mM NaCl. Protein concentration was determined based on the Bradford assay by measuring the absorbance at 595 nm, and subsequently checking against the calibration curve constructed using bovine serum albumin (BSA) as the standard.

### 4.2. Biochemical Analysis of TsBgl

Enzyme activity of TsaBgl was investigated by measuring the formation of *p*-nitrophenol (*p*NP) from the enzymatic hydrolysis of *p*-nitrophenyl-β-d-glucopyranoside (*p*NPG), *p*-nitrophenyl-β-d-cellobioside (*p*NPC), and *p*-nitrophenyl-α-d-glucopyranoside (*p*NPαG) (Sigma-Aldrich, St. Louis, MO, USA). In general, enzyme reactions were performed with 7.5 µg/mL TsaBgl in 100 mM sodium phosphate (pH 6.0) containing 2.5 mM *p*NPG at 55 °C for 5 min in a total volume of 100 µL. Enzyme reactions were terminated by adding 66 µL of 1 M Na_2_CO_3_. The amount of *p*NP produced was measured at 405 nm using a Synergy H1 microplate reader (BioTek Instruments Inc., Winooski, VT, USA) at 25 °C and calculated from a calibration curve using *p*NP as the standard. One unit (U) of enzyme activity was defined as the amount of enzyme required to release 1 μmol of *p*NP per min from 2.5 mM *p*NPG.

To examine the effects of pH on the activity of TsaBgl, enzyme activity was measured in the pH range 4.0–10.0 at 55 °C using a four buffer systems: 100 mM sodium citrate (pH 4.0–6.0), 100 mM sodium phosphate (pH 6.0–7.0), 100 mM Tris-HCl (pH 7.0–9.0), and 100 mM glycine-NaOH (pH 9.0–10.0).

To investigate the effects of temperature on the activity of TsaBgl, the enzyme reaction was examined using 2.5 mM *p*NPG in 100 mM sodium phosphate buffer (pH 7.0) for 5 min at different temperatures ranging from 30 to 70 °C. For thermal stability analysis, residual enzyme activity was measured after pre-incubating TsaBgl at specified temperatures in the absence of substrate. In detail, 7.5 µg/mL TsaBgl was pre-incubated at temperatures ranging from 30 to 80 °C for 10 min in 100 mM sodium phosphate (pH 6.0) before running the enzyme reaction for 5 min by adding 2.5 mM *p*NPG under standard conditions (55 °C and pH 6.0). Relative activity (%) represents the residual enzyme activity relative to that determined without pre-incubation.

To investigate the effects of metal on the activity of TsaBgl, enzyme activity was measured under optimal conditions (55 °C and pH 6.0 in 100 mM sodium phosphate) in the presence of 1 mM of various metal ions, such as LiSO_4_, MgCl_2_, CaCl_2_, MnCl_2_, FeCl_2_, FeCl_3_, CoCl_2_, NiCl_2_, CuCl_2_, ZnCl_2_, CdCl_2_, and CeCl_2_.

Kinetic parameters (*K_m_*, *k_cat_*, and *k_cat_*/*K_m_*) were determined by conducting the enzymatic reaction with 7.5 µg/mL TsaBgl in various concentrations (0 to 10 mM) of *p*NPG at the optimal condition (pH 6.0 and 55 °C). Data fitting was performed using the Michaelis-Menten equation based on SigmaPlot 12.3 software (Systat Software, Erkrath, Germany).

To investigate the tolerance of TsaBgl to Tris and glucose, we performed the enzymatic reaction under the standard conditions and in the absence or presence of different concentrations of Tris (0–210 mM) or glucose (0–830 mM) from 1 M (pH 7.0) or 2 M stock, respectively. In case of Tris inhibition, the enzyme assay was performed at pH 7 (100 mM sodium phosphate) to prevent the potential effect of Tris on pH change in the reaction solution. The relative activity (%) represents the enzyme activity relative to that determined in the absence of Tris or glucose.

### 4.3. Crystallizations

Purified TsaBgl was concentrated to 30 mg/mL using a Centricon filter (Merck; cut-off: 30 kDa). Initial crystallization conditions of TsaBgl were screened using the sitting-drop vapor diffusion method at 22 °C using a Crystal Screen Kit (Hampton Research, Aliso Viejo, CA, USA). Microcrystals were obtained in reservoir solution containing 0.1 M Tris-HCl (pH 8.5), 30% (*w*/*v*) PEG 4000, and 0.2 M MgCl_2_. Crystallization conditions were further optimized by the hanging-drop vapor diffusion method at 22 °C. Rod-shaped TsaBgl crystals were obtained within 1 week by mixing 2 µL protein solution with 2 µL reservoir solution, followed by equilibration against 500 µL of 0.1 M Tris-HCl (pH 7.0), 27% (*w*/*v*) PEG 3350, and 0.2 M MgCl_2_.

### 4.4. X-ray Diffraction Data Collection

X-ray diffraction data were collected on a Beamline 11C at the Pohang Light Source II (PLS-II; Pohang, Korea) [47]. The crystals were transferred into a cryoprotectant solution containing the reservoir solution supplemented with 25% (*v*/*v*) ethylene glycol and then flash-frozen in a liquid nitrogen stream at 100 K. Diffraction images were indexed, integrated, and scaled using HKL2000 [48].

### 4.5. Structure Determination and Analysis

The structure was solved by molecular replacement with MOLPEP [49], using the crystal structure of engineered Bgl from a soil metagenome library (PDB code: 4HZ6) [50] as a search model. The model was manually built using the COOT program [51]. Model refinement was performed using REFMAC5 [52]. The geometry of the final model was evaluated using MolProbity [53]. Structure figures were prepared using PyMOL (DeLano Scientific LLC, San Carlos, CA, USA). Structure-based sequence alignments were created with Clustal-Omega [54] and ESPript [55]. Homologous structures were searched using DALI server [56].

## Figures and Tables

**Table 1:** Effect of metal ions on activity of TsaBgl.

Metal	Relative Activity (%)
None ^a^	100.00 ± 3.05
Li^+^	98.66 ± 2.93
Mg^2+^	97.26 ± 4.83
Ca^2+^	90.80 ± 2.37
Mn^2+^	97.64 ± 5.38
Fe^2+^	69.46 ± 2.43
Fe^3+^	91.02 ± 0.57
Co^2+^	92.23 ± 5.69
Ni^2+^	85.07 ± 3.23
Cu^2+^	8.47 ± 0.50
Zn^2+^	24.63 ± 0.98
Cd^2+^	66.53 ± 4.07
Ce^2+^	96.03 ± 3.89

^a^ None represents the enzymatic reaction in the absence of any metal ion, where the specific activity was 25.9 U/mg.

**Table 2:** Data collection and refinement statistics for TsaBgl.

Data Collection	TsaBgl
Space group	P2_1_2_1_2_1_
Cell dimensions a, b, c (Å)	65.139, 71.293, 99.240
Resolution (Å)	50.0–1.70 (1.73–1.70)
Completeness	98.4 (97.1)
Redundancy	5.7 (4.1)
I/σ(I)	16.50 (2.38)
R_merge_^a^	0.135 (0.423)
CC1/2	0.979 (0.663)
CC*	0.995 (0.893)
Refinement statistics	
Resolution (Å)	49.67–1.70
R_work_ (%) ^b^	14.10
R_free_ (%) ^c^	18.23
B-factor (Averaged)	
Protein	17.37
Tris	29.07
Solvent	33.10
R.m.s deviations	
Bond lengths (Å)	0.013
Bond angles (°)	1.635
Ramachandran plot (%)	
favored	97.06
allowed	2.94

Highest resolution shell is shown in parentheses. ^a^ R_merge_ = Σ*_h_*Σ*_i_*|I*i*(hkl)_<*I*(hkl)>|/Σ*_h_*Σ*_i_*I*_i_*(hkl), where *I_i_*(hkl) is the intensity of the ‘ith’ measurement of reflection hkl and <*I*(hkl)> is the weighted mean of all measurements of hkl. ^b^ R_work_ = Σ||*F*_obs_| − |*F*_calc_||/Σ|*F*_obs_|, where *F*_obs_ and *F*_calc_ are the observed and calculated structure-factor amplitudes, respectively. ^c^ R_free_ was calculated as R_work_ using a randomly selected subset (5%) of unique reflections not used for structure refinement.

**Table 3:** Comparison of kinetic parameters across the GH family 1 Bgls ^a^.

Enzyme	*K_m_* (mM)	*k_cat_* (s^−1^)	*k_cat_*/*K_m_* (mM^−1^ s^−1^)	Optimal Conditions	Reference
TsaBgl	0.36 ± 0.02	18.62 ± 0.32	50.99 ± 2.28	pH 6, 55 °C	This study
TthBgl	0.63	ND ^b^	ND ^b^	pH 6.4, 70 °C	[36]
EanBglA	1.07	32.98	30.8	pH 7, 30 °C	[37]
CceBglA	0.15 ± 0.01	50.67 ± 1.00	340 ± 27	pH 6, 45 °C	[29]
TreBgl2	0.86 ± 0.07	6.91 ± 0.16	8.1 ± 0.8	pH 6, 40 °C	[29]
NkoBgl	0.29 ± 0.02	1.39 ± 0.03	4.8 ± 0.4	pH 5.5, 45 °C	[29]
TmaBglA	0.38 ± 0.02	452.27 ± 8.25	1210 ± 140	pH 6.2, 90 °C	[38]

^a^ Parameters compared here were obtained using *p*NPG as the substrate. ^b^ ND represents “not determined” in the reference.

## Data Availability

The final coordinates and structure factors were deposited in the Protein Data Bank under accession code 7E5J.

## Supplementary Materials

The following are available online, Figure S1: Effect of 1 mM of Tris on the activity of TsaBgl; Figure S2: 2Fo-Fc electron density map of Tris and Tris-binding residues of TsaBgl; Figure S3: Interaction between the N and C Termini of TsaBgl, Figure S4: Superimposition of TsaBgl with other Bgls, Figure S5: Sequence alignment of TsaBgl with other Bgls, Figure S6: Comparison of TsaBgl with other Bgls.

## Sample Availability

Samples of the compounds are not available from the authors.
