# Supplementary material for: Biochemical and Structural Analysis of a Glucose-Tolerant β-Glucosidase from the Hemicellulose-Degrading Thermoanaerobacterium saccharolyticum
Source: Molecules. 2022 Jan 4;27(1):290. doi: 10.3390/molecules27010290 (PMC8746653; doi:10.3390/molecules27010290)
Supplement: Supplementary file 1 [file molecules-27-00290-s001.zip › molecules-1412089-supplementary.pdf]

Supplementary Materials

Biochemical and Structural Analysis of a Glucose-Tolerant β-Glucosidase from the Hemicellulose-Degrading *Thermoanaerobacterium saccharolyticum*

In Jung Kim [1], Uwe T. Bornscheuer [1] and Ki Hyun Nam [1,2,3,*]

[1] Department of Biotechnology and Enzyme Catalysis, Institute of Biochemistry, University of Greifswald, 17489 Greifswald, Germany; ij0308@korea.ac.kr (I.J.K.); uwe.bornscheuer@uni-greifswald.de (U.T.B.)
[2] Department of Life Science, Pohang University of Science and Technology, Pohang 37673, Korea
[3] POSTECH Biotech Center, Pohang University of Science and Technology, Pohang 37673, Korea
* Correspondence: structures@postech.ac.kr; Tel.: +82-10-5208-5730

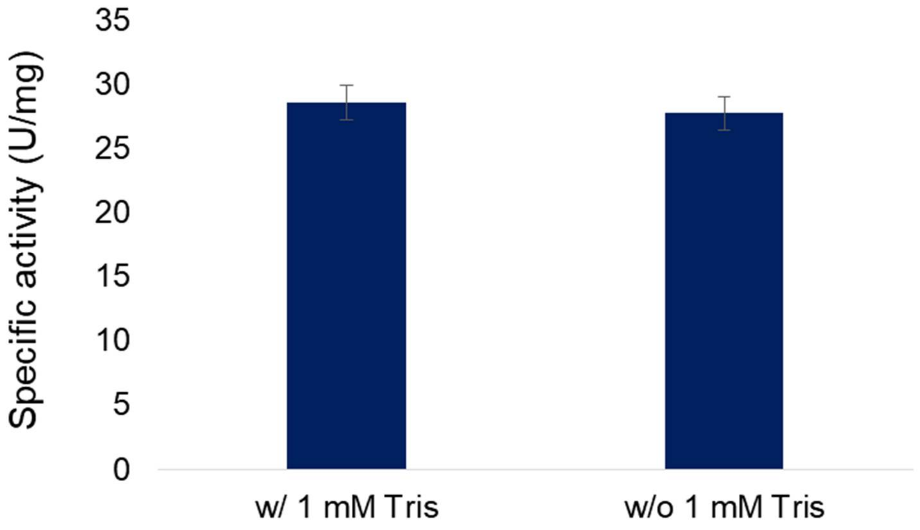

Figure S1. Effect of 1 mM of Tris on the activity of TsaBgl. The enzymatic reaction was performed in the standard condition with or without 1 mM Tris. In case of the reaction without Tris, enzyme stored in 10 mM sodium phosphate buffer (pH 8.0) with 200 mM NaCl was added into the reaction mixture instead of the enzyme stored in 10 mM Tris-HCl buffer with 200 mM NaCl (pH 8.0) (i.e., w/ 1 mM Tris).

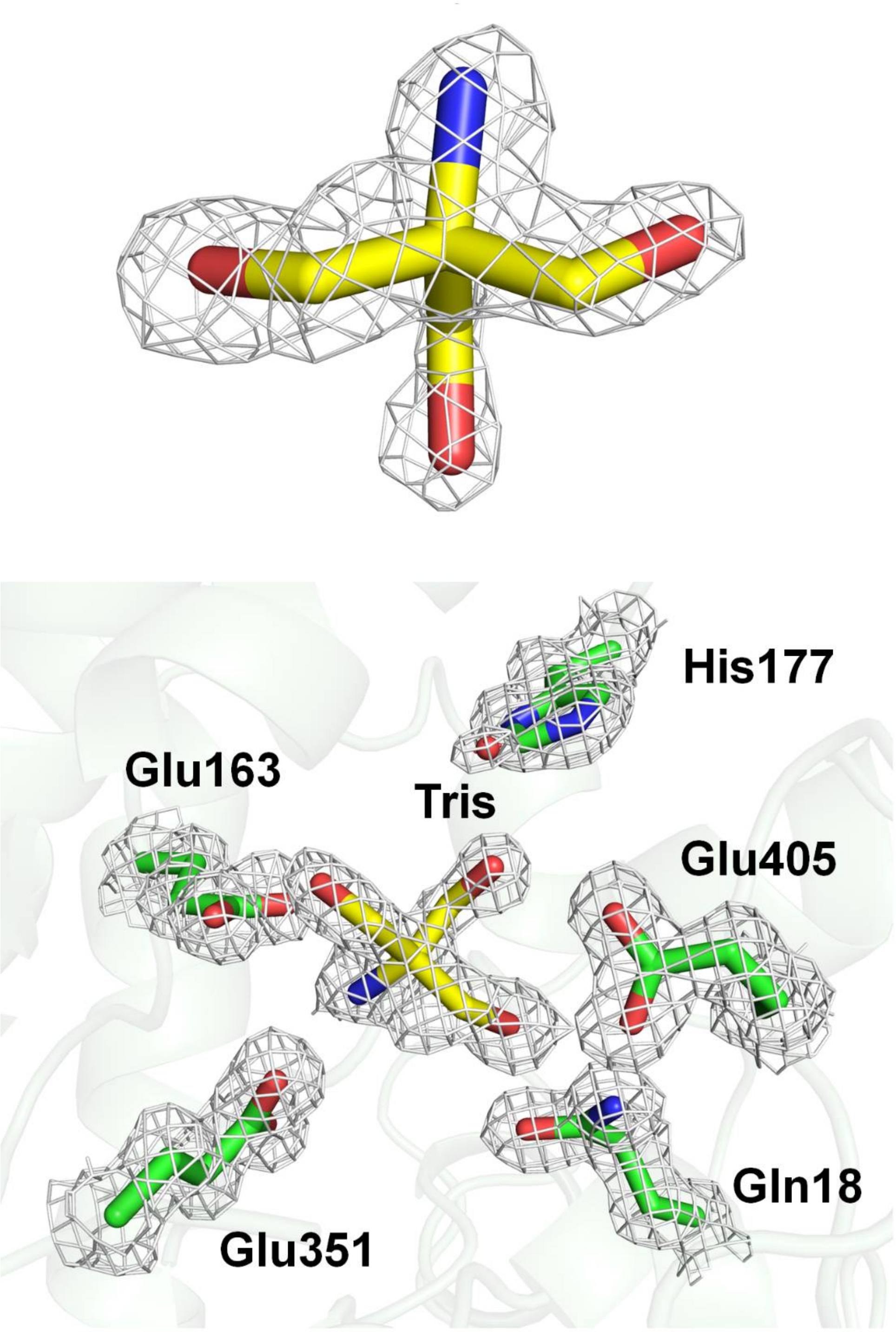

Figure S2. 2Fo-Fc electron density map (grey mesh, contoured at 1.2 σ) of Tris and Tris-binding residues of TsaBgl.

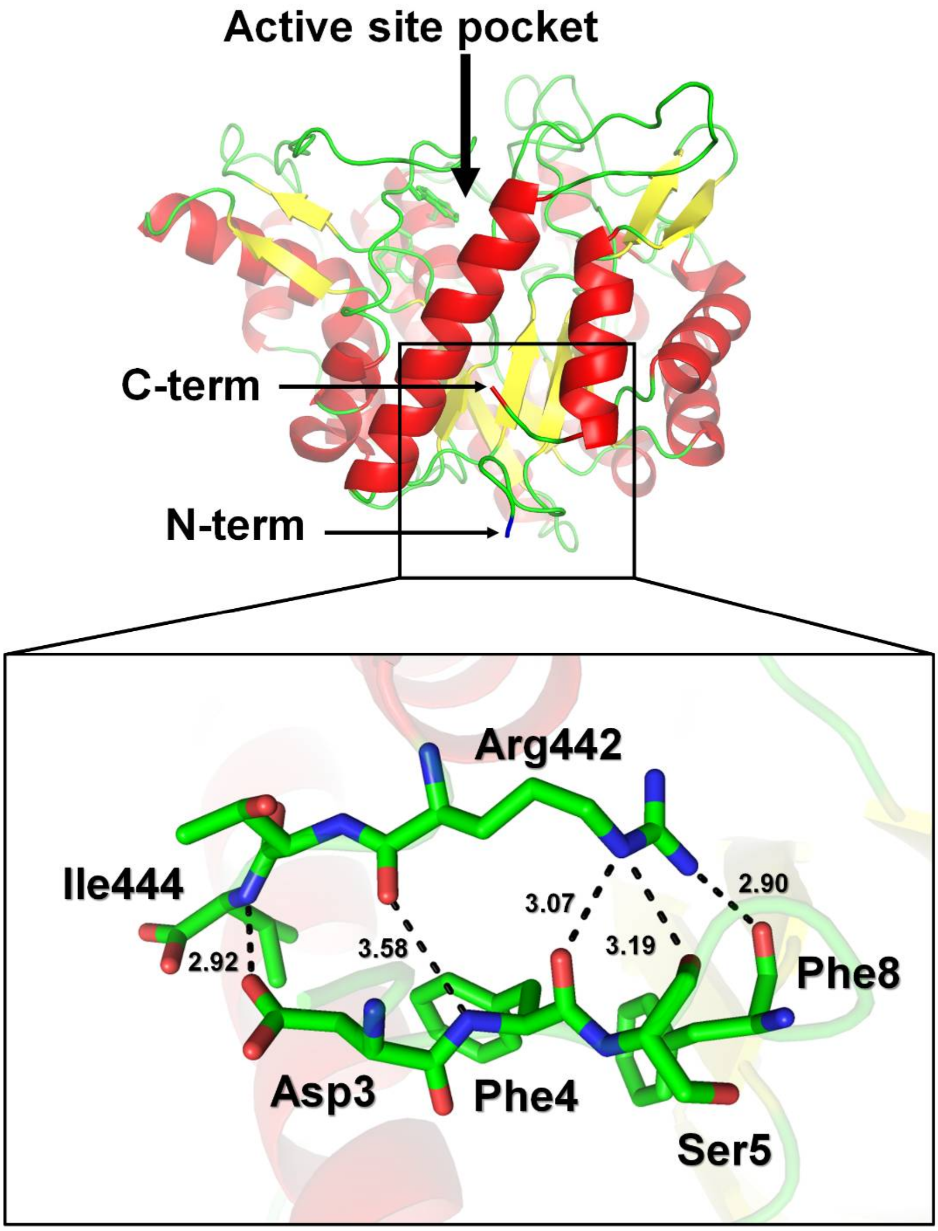

Figure S3. Interaction between the N and C Termini of TsaBgl.

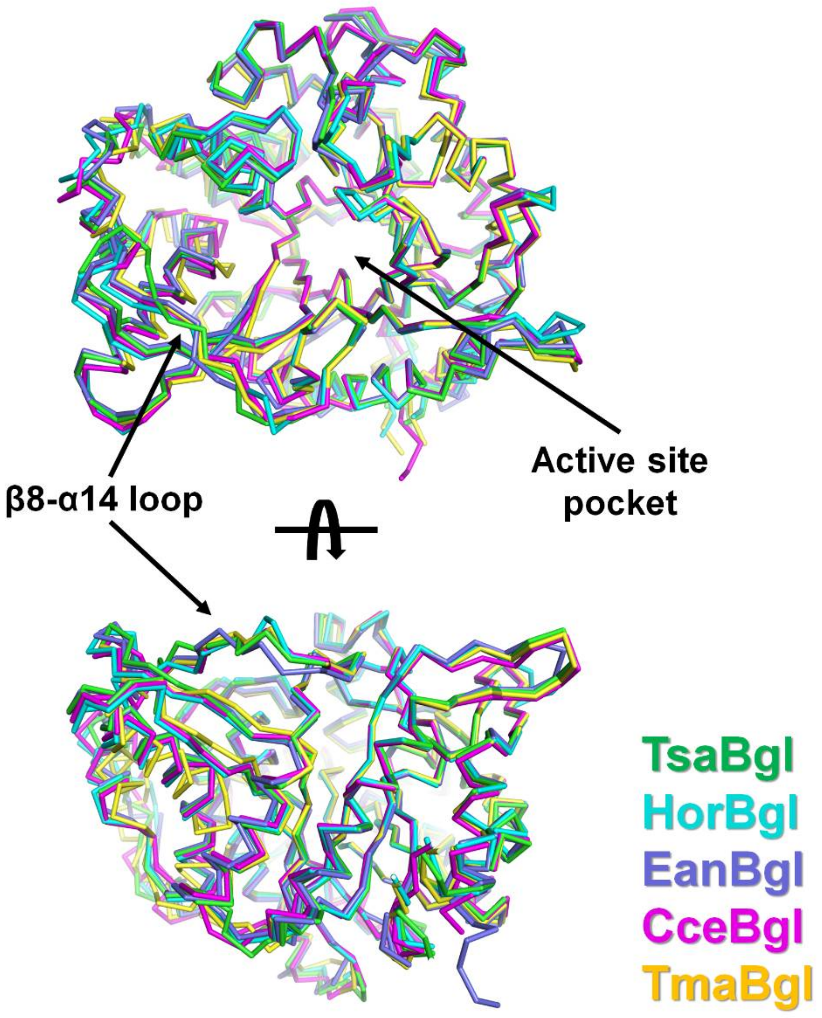

Figure S4. Superimposition of TsaBgl with HorBglB (PDB code: 4PTX), EanBglB (5DT7), CceBglB (3AHX), and TmaBgl (2J79).

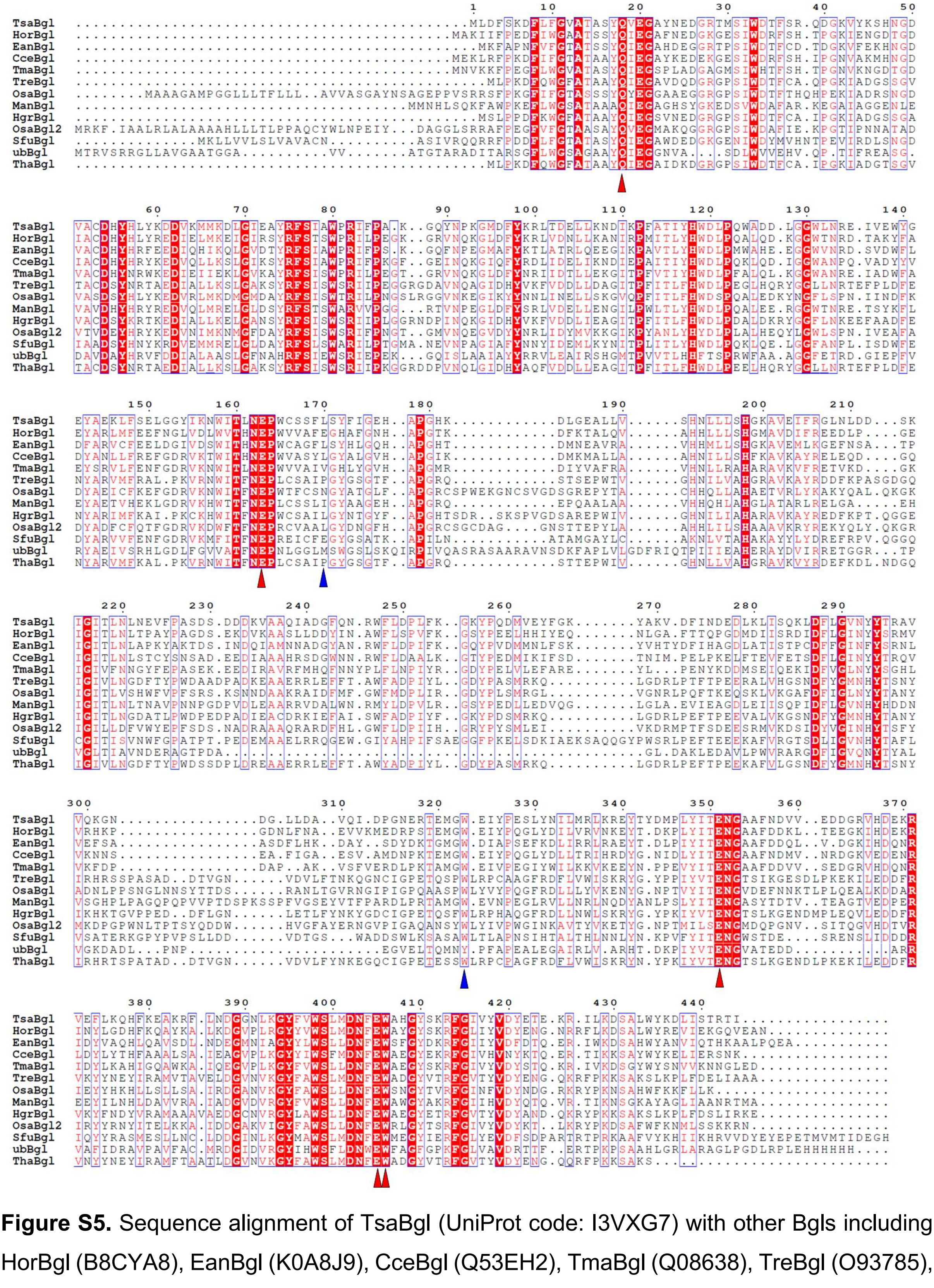

Figure S5. Sequence alignment of TsaBgl (UniProt code: I3VXG7) with other Bgls including HorBgl (B8CYA8), EanBgl (K0A8J9), CceBgl (Q53EH2), TmaBgl (Q08638), TreBgl (O93785), OsaBgl (B8AVF0), ManBgl (B9V8P5), HgrBgl (O93784), OsaBgl2 (A2YPH1), SfuBgl (O61594), ubBgl (A0A5H1ZR35) and ThaBgl (A3FPG4). Residues involved in Tris binding and glucose-tolerance are marked with red and blue triangles, respectively.

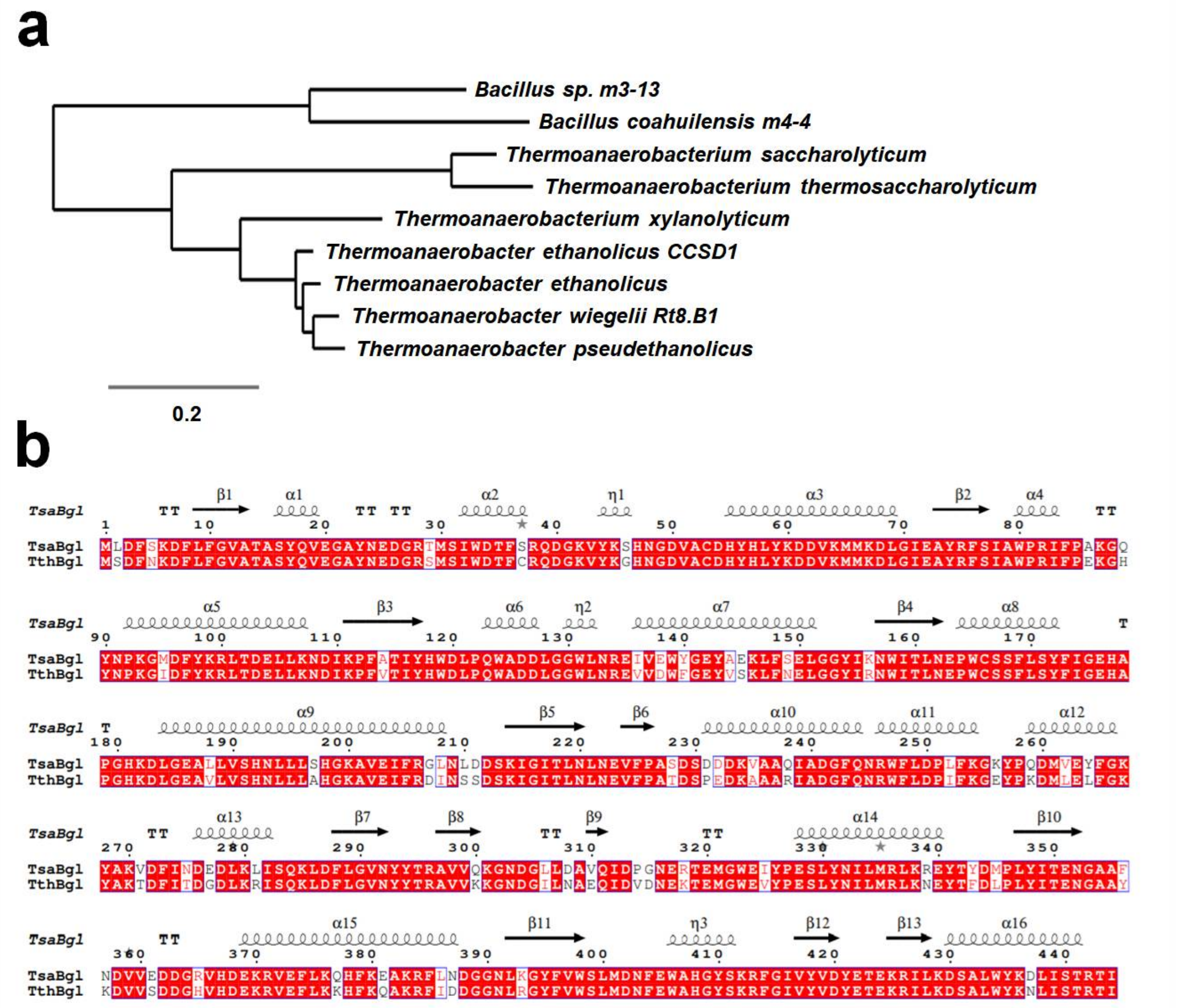

Figure S6. Comparison of TsaBgl with other Bgls. (**a**) Phylogenetic tree analysis of Clade 5 Bgls from TsaBgl (accession number I3VXG7), Bgl from *Thermoanaerobacterium thermosaccharolyticum* (D9TR57), *Thermoanaerobacterium xylanolyticum* (F6BL86), *Thermoanaerobacter pseudethanolicus* (B0KDF9), Thermoanaerobacter wiegelii Rt8.B1(G2MRY3), *Thermoanaerobacter ethanolicus* CCSD1 (C7IQT1), *Thermoanaerobacter ethanolicus* (D3Y2V4), *Bacillus* sp. m3-13 (ZP_07709810.1) and *Bacillus coahuilensis* m4-4 (ZP_03227551.1). (**b**) Sequence alignment of TsaBgl (I3VXG7) and TthBgl (D9TR57).
